# Genomic outbreak investigation of biosafety-level-3 pathogens using nanopore sequencing

**DOI:** 10.1099/mgen.0.001742

**Published:** 2026-06-30

**Authors:** Christine Francesca Thomas, Hanka Brangsch, Herbert Tomaso, Falk Melzer, Gamal Wareth, Jörg Linde

**Affiliations:** 1 Institute of Bacterial Infections and Zoonoses, Federal Research Institute for Animal Health, Friedrich-Loeffler-Institute, Jena, Germany; 2 RNA Bioinformatics and High-Throughput Analysis, Friedrich Schiller University, Jena,07743 Jena, Germany; 3 Institute of Infectious Diseases and Infection Control, Jena University Hospital, Jena, Germany

**Keywords:** *Bacillus anthracis*, *Brucella suis*, *Brucella melitensis*, cgMLST, *Francisella tularensis*, genotyping, nanopore sequencing, outbreak analysis, SNP calling

## Abstract

**Background.** Accurate outbreak analysis is essential for effective infectious disease control. While short-read Illumina sequencing is the current gold standard for genotyping pathogens, Oxford Nanopore Technologies (ONT) offers advantages such as portability and real-time sequencing. However, the accuracy of ONT for single nucleotide polymorphisms (SNP) detection and core genome multilocus sequence typing (cgMLST) remains poorly characterized, which is a critical issue when investigating outbreaks of highly dangerous biosafety-level-3 (BSL-3) agents.

**Aim.** This study evaluates the potential of ONT sequencing for outbreak analysis of four BSL-3 bacterial species with low mutation rates: *Bacillus (Ba.) anthracis, Brucella (Br.) melitensis, Brucella (Br.) suis* and *Francisella (F.) tularensis*. Strains originating from epidemiologically defined outbreaks, with existing Illumina sequencing data, were selected for analysis. SNP calling was evaluated using three analytical strategies (PACU, clair3 and an assembly-based approach with snippy). The resulting genomic clusters were compared to epidemiologically defined outbreaks that had previously been reproduced using Illumina sequencing. Additionally, cgMLST was performed to evaluate genotyping resolution across the sequencing platforms.

**Results.** Minor discrepancies in strain clustering were observed, particularly for *F. tularensis*, where homopolymeric regions contributed to false positives in read-based callers. The assembly-based snippy approach achieved the highest F1 score (0.96–0.99) across all species, followed closely by *ab initio* SNP callers PACU (F1 : 0.88–0.97) and clair3 (F1 : 0.83–0.98). Minor discrepancies in strain clustering were observed, particularly for *F. tularensis*, where homopolymeric regions contributed to false positives in read-based callers. CgMLST analysis showed high concordance between ONT and Illumina for *Brucella* spp., but greater variability for *Ba. anthracis* and *F. tularensis*.

**Conclusions.** ONT sequencing, particularly when used with ONT assembly-based SNP calling, enables reliable outbreak analysis of highly pathogenic, low-diversity bacteria. While challenges remain for specific species and genomic features, ONT is a promising alternative for high-resolution bacterial genotyping in outbreak scenarios.

**Impact Statement**

This study provides a species-level evaluation of ONT sequencing for high-resolution genotyping of four low-diversity biosafety-level-3 bacterial pathogens. By evaluating three ONT-based analytical strategies for SNP-based outbreak reconstruction (PACU, clair3 and an assembly-based snippy workflow) against epidemiologically defined outbreaks that had previously been reproduced using Illumina sequencing, we demonstrate that ONT, while not error-free, can achieve SNP-level accuracy sufficient for reliable outbreak reconstruction when appropriate analysis strategies and thresholds are applied. The assembly-based approach showed the highest concordance, highlighting the benefit of consensus-based error reduction prior to SNP calling, whereas read-based callers exhibited species-dependent limitations, particularly error inflation in homopolymeric regions and hence decreased precision in *Francisella (F.) tularensis*. Similarly, cgMLST derived from ONT assemblies showed high agreement with Illumina across *Brucella* spp. and acceptable divergence in *Bacillus (Ba.) anthracis* and *F. tularensis*, with discrepancies representing platform-specific assembly artefacts rather than systematic ONT failure. These results clarify the operational boundaries of ONT for fine-scale genomic epidemiology and highlight that, despite known susceptibility to homopolymer-associated errors, ONT sequencing can provide sufficiently accurate data for outbreak detection in multiple pathogens. By defining both the capabilities and the remaining limitations of ONT for SNP- and cgMLST-based typing, this study contributes essential technical guidance for integrating ONT into real-time pathogen genomics pipelines, particularly in contexts where rapid, portable or resource-efficient sequencing is required.

## Data Summary

The sequencing data has been deposited with the European Nucleotide Archive and is available under Bioproject PRJEB94163 (https://www.ebi.ac.uk/ena/browser/view/PRJEB94163). Detailed accession numbers for all strains are available in Supplement Table S3. *Bacillus anthracis* Illumina sequencing data is available under Bioproject PRJNA656733 (https://www.ebi.ac.uk/ena/browser/view/PRJNA656733). *Francisella tularensis* Illumina sequencing data is available under Bioproject PRJEB40963 (https://www.ebi.ac.uk/ena/browser/view/PRJEB40963). *Brucella suis* Illumina sequencing data is available under Bioproject PRJEB61768 (https://www.ebi.ac.uk/ena/browser/view/PRJEB61768).

## Introduction

Whole-genome sequencing (WGS) has fundamentally transformed genotyping in pathogenic bacteria by enabling unprecedented resolution in the analysis of genetic relationships [1]. Traditional methods such as multilocus sequence typing (MLST) and multiple-locus variable number of tandem repeat analysis, while useful, offer limited discriminatory power for outbreak investigation [2,3]. In contrast, WGS supports high-resolution genotyping approaches such as core genome multilocus sequence typing (cgMLST) and core genome single-nucleotide polymorphism (cgSNP) typing, which allow the fine-scale differentiation of closely related isolates and the detailed reconstruction of transmission events [4,6]. These methods have become central to modern bacterial surveillance and outbreak analysis due to their precision and ability to be standardized across laboratories [7]. However, SNP-based analyses remain technically demanding, particularly for pathogens with low mutation rates, where isolates may differ by only a small number of nucleotides [8]. In such cases, even minimal sequencing errors or insufficient data quality can significantly distort phylogenetic interpretation, highlighting the critical importance of accurate sequencing and robust bioinformatics pipelines [9].

Illumina sequencing is a well-established method for genotyping, offering low error rates and high base precision, which enables reliable outbreak investigations [10]. SNP calling pipelines like snippy are commonly used to identify SNPs and generate cgSNP alignments for phylogenetic analysis [11,12]. In contrast, Oxford Nanopore Technologies (ONT) sequencing, while exhibiting higher error rates (∼3–5%) [13], provides advantages in resolving complex genomic regions due to the generation of long reads. Sequencing using ONT has become increasingly valuable in bacterial epidemiology, offering rapid sequencing capabilities that enable real-time monitoring and pathogen identification, which are essential advantages for controlling infectious disease outbreaks [14,21]. In addition, the comparatively low cost and portability of ONT devices make them an attractive option for laboratories in lower resource settings. This is particularly relevant for genomic surveillance of high-consequence pathogens in resource-limited settings, where rapid and portable sequencing is advantageous but still requires sufficient resolution for outbreak discrimination.

Multiple studies have shown that cgMLST analyses based on ONT sequencing for bacterial species such as *Neisseria gonorrhoeae* [22], *Bordetella pertussis* [14] or *Corynebacterium* (*C.*) *diphtheriae* [20] correctly differentiate between the strains. However, a study by Lohde *et al*. [15] investigating outbreak data of *Klebsiella* (*K*.) *pneumoniae* detected differences between Illumina and ONT sequencing data and identified methylation as a main source of errors in ONT reads when applying cgMLST analysis. This was confirmed by Dabernig-Heinzig *et al*. [23]*,* who evaluated nanopore sequencing for cgMLST across four human bacterial pathogens in five labs, revealing strain-specific typing errors linked to methylated DNA. In line with Lohde *et al*. [15], Neuenschwander *et al*. [20] proposed a PCR-based library preparation for ONT to avoid methylation that could influence basecalling, thereby improving cgMLST accuracy in vancomycin-resistant *Enterococci*. In a first attempt to assess the accuracy of ONT sequencing on biosafety-level-3 (BSL-3) bacteria, Linde *et al*. [9] compared ONT sequencing data generated with R9.4 and R10.4.0 flowcells to Illumina sequencing data regarding genetic marker detection and genotyping of *Bacillus* (*Ba*.), *Brucella* (*Br*.) ssp. and *Francisella* (*F*.). Here, ONT showed promising cgMLST resolution for some species [9]. In contrast, SNP-based outbreak reconstruction using ONT data has been less extensively evaluated, particularly for highly clonal bacterial pathogens.

SNP-based phylogenetic analyses at single-nucleotide level, which are critical for resolving highly clonal outbreaks, have not yet been fully validated for ONT across diverse bacterial species. A recent study by Hall *et al*. [24] has demonstrated the superiority of deep learning-based variant callers such as clair3 in improving SNP detection from ONT data. As highlighted by a study from Belgium, appropriate filtering can markedly enhance data quality and reliability by accounting for these platform-specific biases, resulting in the pipeline Prokaryotic Awesome variant Calling Utility (PACU) [16]. This is particularly relevant for bacteria with low mutation rates, where even a single SNP can significantly impact phylogenetic conclusions [8]. To address this gap, four bacterial species were selected: *Ba. anthracis*, *Br. melitensis*, *Br. suis* and *F. tularensis*. These species are highly pathogenic zoonotic bacteria that cause severe notifiable zoonotic diseases [25]. *Br. suis* infects swine and humans [26]; *Br. melitensis*, the most virulent *Brucella* sp. for humans, affects cattle, goats and sheep [27]; *Ba. anthracis* can cause anthrax, primarily in herbivores, with humans as incidental hosts [28]; and *F. tularensis* causes tularemia in a wide range of mammals, including humans [4]. Most of these pathogens are classified as BSL-3 biological agents because they can cause severe human disease, have zoonotic potential, can be transmitted via aerosols and have limited treatment or vaccine options. As they have been used in bioterrorism, detailed knowledge of the genotypes occurring in different regions worldwide is needed. The genomes of all four species discussed in this manuscript are considered stable, meaning there is minimal genetic variation between strains, even when comparing strains from geographically distant regions [29,31].

In this study, we evaluate the potential of ONT sequencing for outbreak analysis of highly pathogenic bacteria, with a particular focus on validating SNP calling and cgMLST accuracy. Four critical pathogens, *Br. suis, Br. melitensis, Ba. anthracis* and *F. tularensis*, were selected, each represented by three genome-based clusters containing two to three closely related strains. To assess the accuracy of SNP-based outbreak reconstruction using ONT data, three analytical strategies were employed (clair3, PACU and an assembly-based snippy workflow). Epidemiologically defined outbreaks that had previously been reproduced using Illumina sequencing served as reference for benchmarking. Sequencing data were assembled and analysed for cgMLST using organism-specific cgMLST schemes. Subsequent clustering analyses were conducted to evaluate the capacity of ONT sequencing, combined with cgMLST and SNP-based approaches, to reliably resolve outbreak-related isolates and support high-resolution bacterial genotyping.

## Methods

### Strain selection

#### Outbreak analysis

To investigate the performance of high-resolution genotyping, for four highly pathogenic bacteria (*Ba. anthracis*, *Br. melitensis, Br. suis* and *F. tularensis*) known outbreaks were analysed (Table 1). The outbreaks, as defined by the corresponding national reference laboratory, were confirmed by studies based on Illumina data; *Ba. anthracis* sequences are publicly available within the BioProject PRJNA656733, *Br. melitensis* within the BioProject PRJEB71735, *Br. suis* within the BioProject PRJEB61768 and *F. tularensis* within the BioProject PRJEB40963). Reference genomes were retrieved from NCBI RefSeq [32]. For *Bacillus anthracis,* Ames Ancestor (reference genome: GCF_000008445.1), *Brucella melitensis* M28 (reference genome: GCF_000192725.1), *Brucella suis* 1330 (reference genome: GCF_000007505.1) and *Francisella tularensis* FSC200 (reference genome: GCF_000168775.2).

**Table 1.** Outbreak strain selection with accession numbers for ONT data and Illumina data as well as the corresponding literature

Strain	Region	Cluster	Species	Literature	Run accession no. ONT	Run accession no. Illumina
13T0009	Soest	C1-FraTu	*F. tularensis*	Linde *et al*. [4]	ERR15335129	ERR5100391
17T1131	Soest	C1-FraTu	*F. tularensis*	Linde *et al*. [4]	ERR15335130	ERR5100514
19T0042	Soest	C1-FraTu	*F. tularensis*	Linde *et al*. [4]	ERR15335132	ERR5100568
13T0036	Ludwigsburg	C2-FraTu	*F. tularensis*	Linde *et al*. [4]	ERR15335132	ERR5100393
14T0026	Ludwigsburg	C2-FraTu	*F. tularensis*	Linde *et al*. [4]	ERR15335133	ERR5100407
15T0767	Ludwigsburg	C2-FraTu	*F. tularensis*	Linde *et al*. [4]	ERR15335134	ERR5100472
14T0114	Hannover	C3-FraTu	*F. tularensis*	Linde *et al*. [4]	ERR15335135	ERR5100427
16T0026	Hannover	C3-FraTu	*F. tularensis*	Linde *et al*. [4]	ERR15335136	ERR5100493
18T0200	Hannover	C3-FraTu	*F. tularensis*	Linde *et al*. [4]	ERR15335137	ERR5100548
14RA5914	Dobichau	C1-BaAn	*Ba. anthracis*	Abdel-Glil *et al*. [6]	ERR15335117	SRR12435818
14RA5915	Dobichau	C1-BaAn	*Ba. anthracis*	Abdel-Glil *et al*. [6]	ERR15335118	SRR12435815
14RA5916	Dobichau	C1-BaAn	*Ba. anthracis*	Abdel-Glil *et al*. [6]	ERR15335119	SRR12435814
12RA1944	Stendal	C2-BaAn	*Ba. anthracis*	Abdel-Glil *et al*. [6]	ERR15335115	ERR10820686
12RA1947	Stendal	C2-BaAn	*Ba. anthracis*	Abdel-Glil *et al*. [6]	ERR15335116	ERR15342670
09RA5721	Bavaria	C3-BaAn	*Ba. anthracis*	Abdel-Glil *et al*. [6]	ERR15335112	ERR15342667
22RA24624	Bavaria	C3-BaAn	*Ba. anthracis*	Abdel-Glil *et al*. [6]	ERR15335114	ERR15342669
21RA23352	Bavaria	C3-BaAn	*Ba. anthracis*	Abdel-Glil *et al*. [6]	ERR15335113	ERR15342668
14RB8412	Mecklenburg	C1-BrSu	*Br. suis*	Melzer *et al*. [43]	ERR15335126	ERR11526628
15RB2242	Biberach	C1-BrSu	*Br. suis*	Melzer *et al*. [43]	ERR15335127	ERR11526630
15RB2997	Biberach	C1-BrSu	*Br. suis*	Melzer *et al*. [43]	ERR15335128	ERR15342675
08RB3701	Mecklenburg	C2-BrSu	*Br. suis*	Melzer *et al*. [43]	ERR15335121	ERR11526645
08RB3277	Mecklenburg	C2-BrSu	*Br. suis*	Melzer *et al*. [43]	ERR15335122	ERR11526592
08RB3450	Mecklenburg	C2-BrSu	*Br. suis*	Melzer *et al*. [43]	ERR15335120	ERR15342671
17RB15632	Mecklenburg	C3-BrSu	*Br. suis*	Melzer *et al*. [43]	ERR15335123	ERR15342672
17RB15633	Mecklenburg	C3-BrSu	*Br. suis*	Melzer *et al*. [43]	ERR15335125	ERR15342674
17RB15634	Mecklenburg	C3-BrSu	*Br. suis*	Melzer *et al*. [43]	ERR15335124	ERR15342673
21RB23838	Istanbul	C1-BrMel	*Br. melitensis*	Akar *et al*. [42]	ERR15335106	ERR15342661
21BR23839	Istanbul	C1-BrMel	*Br. melitensis*	Akar *et al*. [42]	ERR15335109	ERR15342664
21RB23844	Sanliurfa	C1-BrMel	*Br. melitensis*	Akar *et al*. [42]	ERR15335104	ERR15342659
21RB23854	Maras	C2-BrMel	*Br. melitensis*	Akar *et al*. [42]	ERR15335105	ERR15342660
21RB23851	Maras	C2-BrMel	*Br. melitensis*	Akar *et al*. [42]	ERR15335108	ERR15342663
21RB23871	Sanliurfa	C2-BrMel	*Br. melitensis*	Akar *et al*. [42]	ERR15335107	ERR15342662
21RB23785	Adiyaman	C3-BrMel	*Br. melitensis*	Akar *et al*. [42]	ERR15335110	ERR15342665
21RB23800	Aydin	C3-BrMel	*Br. melitensis*	Akar *et al*. [42]	ERR15335111	ERR15342666

The sequencing data (ONT data of all strains and Illumina data of *Br. melitensis*) has been deposited with the European Nucleotide Archive and is available under Bioproject PRJEB94163 (https://www.ebi.ac.uk/ena/browser/view/PRJEB94163). *Ba. anthracis* Illumina sequencing data is available under bioproject PRJNA656733 (https://www.ebi.ac.uk/ena/browser/view/PRJNA656733). *F. tularensis* Illumina sequencing data is available under bioproject PRJEB40963 (https://www.ebi.ac.uk/ena/browser/view/PRJEB40963). *Br. suis* Illumina sequencing data is available under Bioproject PRJEB61768 (https://www.ebi.ac.uk/ena/browser/view/PRJEB61768).

### Cultivation and sequencing

#### Cultivation and DNA extraction

The strains were obtained from the corresponding German national reference laboratories for anthrax, brucellosis and tularaemia, respectively, hosted by the Friedrich-Loeffler-Institute. To minimize biological variation arising from spontaneous mutations, strains were obtained directly from the original stocks provided by the respective reference laboratories. No additional subculturing beyond the minimal propagation required for DNA extraction was performed, in order to reduce the risk of accumulating SNPs. *Ba. anthracis* strains were cultivated on nutrient agar (Merck, Darmstadt, Germany) for 24 h at 37 °C, and DNA was extracted using the Genomic-tip 100 G^−1^ kit (Qiagen, Hilden, Germany). *Brucella* sp. (*Br. melitensis* and *Br. suis* biovar 2) were grown on nutrient agar with 7.5% calf blood for 48 h at 37 °C. DNA was isolated from inactivated biomass of the four species with the NucleoBond HMW DNA kit (MACHEREY–NAGEL, Düren, Germany) for high-molecular DNA. The nine *F. tularensis* strains were cultivated on cysteine heart agar (CHA, Becton Dickinson, BD Heidelberg, Germany) at 37 °C with 5% CO_2_ for 72 h. The DNA used for WGS was prepared using the QIAGEN Genomic-tip 20 G^−1^ Kit (Qiagen GmbH, Hilden, Germany). The DNA extraction was performed according to the instructions of the manufacturer for sample preparation and the lysis protocol for bacteria using 1 ml buffer B1 with 2 µl RNase A, 45 µl proteinase K and 20 µl lysozyme.

#### Sequencing

All 34 outbreak strains were sequenced with a MinION Mk1B from ONT (Oxford, United Kingdom). Nanopore sequencing library preparation and barcoding was done using the Native Barcoding Kit 24 V14 (SQK-NBD114.24). The sequencing was performed with a R10.4.1 flowcell (FLO-MIN114) according to the manufacturer’s instructions. The sequencing data have been deposited with the European Nucleotide Archive and are available under Bioproject PRJEB94163.

### Bioinformatics analysis

#### Basecalling

For ONT sequencing data, basecalling was performed with dorado v1.0.0 and model dna_r10.4.1_e8.2_400bps_sup@v5.2.0 [33].

#### SNP calling

**Fig. 1.**
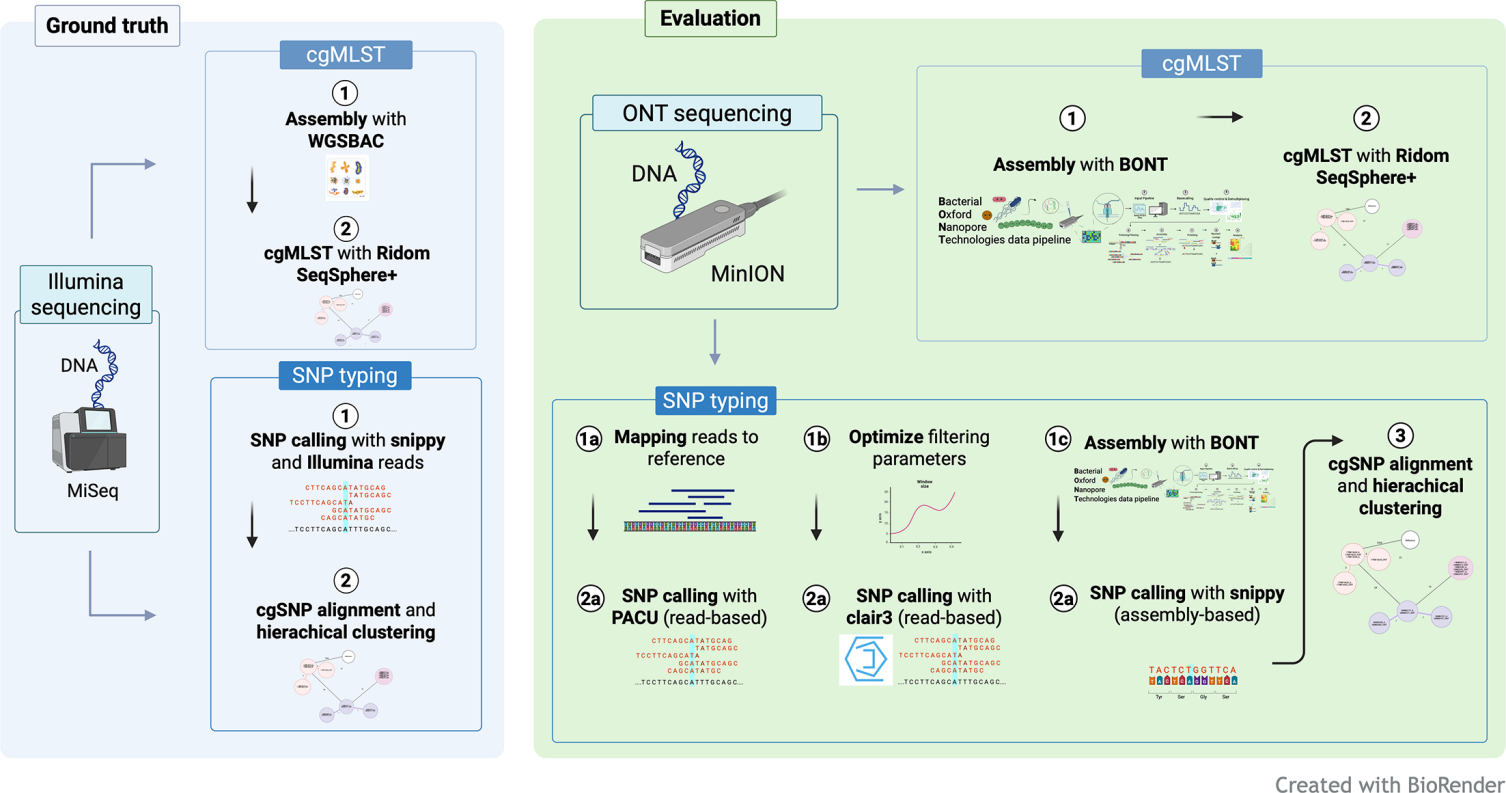
Study design for evaluating genotyping methods with ONT sequencing data. The DNA of the different species (*Ba. anthracis*, *Br. melitensis*, *Br. suis* and *F. tularensis*) was sequenced with Illumina and ONT. Downstream analysis was performed according to the sequencing technology.

Different published variant calling software were used to assess the utility of ONT sequencing data for SNP calling. If not mentioned differently, default settings were applied for each tool. For ONT sequencing data (basecalled with both dorado versions), three different methods were evaluated (Fig. 1).

First, PACU [16] (https://github.com/BioinformaticsPlatformWIV-ISP/PACU) was used. Here, the chromosomes and plasmids of the reference genomes for *Ba. anthracis* and *Brucella* spp. were concatenated as PACU does only accept one contig for the reference sequence.

Second, clair3 [34] was used to call variants, based on a study by Hall *et al*. [24], which identified deep learning tools such as clair3 as the best performing variant callers for ONT sequencing data of bacteria. Based on filtering parameter optimization, clair3 was applied with the model r1041_e82_400bps_sup_v430_bacteria_finetuned with the best performing filters (allele frequency>0.66, coverage depth>22, quality of call>7 and window size=12 bps). To ensure comparability, the cgSNP alignment was built the same way as PACU. The workflow applied for clair3 variant calling can be accessed here: https://gitlab.com/ChristineThms/BONSai, v1.0.

Third, snippy based on polished assemblies was used. Snippy is a variant caller for short-read data but also accepts assemblies as an input. Therefore, assemblies were built using the pipeline BONT (v1.0.0, https://gitlab.com/FLI_Bioinfo/BONT). The basecalled files (with v5.2.0 and dorado 1.0.0) were filtered with filtlong [35] (with --target_bases 500000000, --min_length 800 --keep_percent 90), assembled using flye [36], and polished with medaka [37] using the model r1041_e82_400bps_bacterial_methylation. These assemblies were used together with snippy v4.6.0 [12] (--contigs option) to identify SNPs based on assemblies. The cgSNP alignment was built with snippy-core.

Illumina sequencing data from the published outbreaks were processed with snippy v4.6.0 [12] to identify SNPs. The cgSNP alignment was built with snippy core and snp-dists v0.8.2 was used to compute distances between strains. To identify groups of similar items within the datasets, hierarchical clustering was performed. First, pairwise dissimilarities were calculated using the squareform representation of the distance matrices. Then, the linkage function from the SciPy library was used to generate hierarchical cluster trees using the average linkage method, the fcluster function was applied to each dendrogram. The cluster threshold was defined as five allelic differences for all species. Graphs (minimum spanning trees) were generated using BioRender, and the clustering threshold was set to five allelic differences across all species.

#### Optimization of filtering parameters

We performed filtering parameter optimization for clair3 for the following parameters: read length, quality of reads, allele frequency, coverage depth, quality of call, strand bias and a window size within SNPs were filtered out. First, for each species, we optimized each filtering parameter to maximize the concordance between SNPs called from ONT data and those from Illumina sequencing data. The optimization is therefore based on the F1 score of clair3 applied with the specific parameter.

Precision (proportion of called ONT SNPs that were Illumina SNPs), recall (proportion of Illumina SNPs that were called for ONT) and F1-score (harmonic mean of precision and recall) were calculated as follows:


$$\left(1\right)Precision=\frac{TP}{TP+FP}$$

$$\left(2\right)Recall=\frac{TP}{TP+FN}$$

$$\left(3\right)F1=2*\frac{Precision*Recall}{Precision+Recall}$$

where *TP* is the number of true positives, *FP* is the number of false positives and *FN*

is the number of false negatives. Let θ∈D⊂R be the filtering threshold for a given parameter. The domain D is refined according to the results from the parameter-specific optimization. The optimization objective is to find the optimal threshold $\theta^{*}$ that maximizes the F1 score:


$$\left(4\right)\;\;\;\;\theta^{*}=\underset{\left\{\theta \in D\right\}}{\arg}\max\;F1\left(\theta \right)$$

Afterwards, we excluded all parameters not influencing the F1 score (or only reducing F1) and optimized the resulting parameters together and across all species, including the ARI, to ensure that not only precision is maximized but also cluster concordance.


$$\left(5\right)ARI=\frac{\sum_{ij}(\begin{array}{c} n_{ij}\\ 2 \end{array})-\frac{\sum_{i}\left(\begin{array}{c} a_{i}\\ 2 \end{array}\right)\sum_{j}\left(\begin{array}{c} b_{j}\\ 2 \end{array}\right)}{\left(\begin{array}{c} n\\ 2 \end{array}\right)}}{\frac{1}{2}\left(\sum_{i}\left(\begin{array}{c} a_{i}\\ 2 \end{array}\right)+\sum_{j}\left(\begin{array}{c} b_{j}\\ 2 \end{array}\right)\right)-\frac{\sum_{i}\left(\begin{array}{c} a_{i}\\ 2 \end{array}\right)\sum_{j}\left(\begin{array}{c} b_{j}\\ 2 \end{array}\right)}{\left(\begin{array}{c} n\\ 2 \end{array}\right)}}$$

where $a_{i}=\sum_{j}n_{ij}$and$b_{j}=\sum_{i}n_{ij}$, and $\left(\begin{array}{c} n\\ 2 \end{array}\right)=\frac{n(n-1)}{2}$ denotes the number of pairs.

The optimal parameter vector $\theta^{*}$ is then defined as the one that maximizes a weighted combination of the F1 score and the ARI score:


$$\left(6\right)\;\theta^{*}=\underset{\left\{\theta \in D\right\}}{\arg}\max\;0.5\;*F1\left(\theta \right)+0.5\;*ARI\left(\theta \right)$$

which is solved again using Bayesian optimization, implemented in *gp_minimize* from the *scikit-optimize* library with 10 calls per batch. The optimization is stopped if the best scores from the last five batches did not differ in 0.01. The domain *D* is refined according to the results from the parameter-specific optimization.

#### Performance assessment of variant calling methods

Each method was initially evaluated for its ability to correctly identify SNPs relative to Illumina-based calls. Specifically, precision, recall and F1 score for each method Formula (1), (2), and (3) were used to evaluate the performance. To quantify clustering agreement, we used the ARI, which measures similarity between two clusters [see Formula (5)].

#### cgMLST

To assess the performance of cgMLST, assemblies were first created for all strains. Consequently, ONT reads were assembled using the pipeline BONT (v1.0.0, https://gitlab.com/FLI_Bioinfo/BONT). The basecalled files (with v5.2.0 and dorado 1.0.0) were filtered with filtlong [35] (with --target_bases 500000000, --min_length 800 --keep_percent 90, assembled using flye [36], and polished with medaka [37] using the model r1041_e82_400bps_bacterial_methylation.

Illumina reads were assembled using the pipeline WGSBAC (v 2.2.3) [38]. Here, the raw paired-end reads served as input, the coverage was calculated, and the quality of the short-read sequencing data was assessed with FastQC [39]. A mean coverage >30 fold was considered acceptable for further analyses. The quality and adapter trimming of the Illumina reads and assembly were performed by Shovill v. 1.0.4 719 [40].

The cgMLST analysis was performed using Illumina and ONT assemblies and the corresponding reference genomes with the software SeqSphere+ [41] and the respective schemes for *Ba. anthracis* [6], *Brucella* spp. [5] and *F. tularensis* [4]. The cluster threshold was defined as five allelic differences for all species. Graphs (minimum spanning trees) were generated using BioRender, and the clustering threshold was set to five allelic differences across all species.

## Results

### Strain selection

To validate SNP-based analysis approaches, we selected strains from well-characterized outbreaks. The epidemiological background of the strains in connection with other strains of the species can be found in the corresponding references.

For *Ba. anthracis,* strains from outbreaks in the Bavarian Alps (Germany) and central Germany (strains 12RA1944, 21RA1947, 14RA5914, 14RA5915 and 14RA5916), isolated between 2009 and 2021 were included.

For *Br. melitensis*, eight outbreak isolates from Türkiye were chosen [42], that were originally attributed to three different outbreak clusters (cluster 1 : 21RB23844, 21RB23854, 21RB23838; cluster 2: 21RB23871, 21RB23851, 21RB23839; and cluster 3 : 21RB23785, 21RB23800).

Further, nine *Br. suis* isolates were selected. These were previously shown to be involved in outbreaks in domestic pigs (*Sus scrofa domesticus*) and wild boar (*Sus scrofa*) in Germany [43] and had been isolated between 2008 and 2017, mostly in Mecklenburg-Western Pomerania.

Lastly, nine isolates from three geographically distinct tularemia outbreaks in Germany involving European brown hares (*Lepus europaeus*) and *F. tularensis* were included in this study. These outbreaks occurred between 2013 and 2019 in North Rhine-Westphalia (13T0009 in 2013, 17T1131 in 2017 and 19T0042 in 2019), Baden-Wuerttemberg (13T0036 in 2013, 14T0026 in 2014 and 15T0767 in 2015) and Lower Saxony (14T0114 in 2014, 16T0026 in 2016, 18T0200 in 2018). All isolates originated from European brown hares.

### Evaluation strategy for SNP calling

We evaluated three distinct SNP-based analytical approaches using ONT sequencing data to assess their performance across species. The rationale for selecting these strategies was to represent the range of commonly and recently applied strategies for SNP detection on ONT data: PACU, clair3, and assembly-based snippy workflow (Table 2). For the assembly-based approach, reads are artificially generated from the assemblies and then analysed with snippy following the same procedure used for read-based snippy. From all 34 sequenced strains, sufficient high-quality sequencing data were generated, as displayed by a minimum coverage of 31-fold, a minimum mean read length of 1,124 bps and at least 75.2% of reads reaching Q15 (Table S1, available in the online Supplementary Material).

**Table 2.** Workflow and applied filters of evaluated SNP calling pipelines for ONT sequencing data

Tool	PACU	Clair3	Snippy
Type of data used	Basecalled ONT reads	Basecalled ONT reads	Artificial Illumina-like reads generated from ONT assembly
SNP caller	bcftools pileup	Neural-network-based caller +minimap2	BWA MEM +freebayes
Filtering	allele frequency (AF) >0.9, depth (DP) <5, quality of call >25, window size of 10 SNPs, and for mapping quality >5, low coverage regions will be excluded <5-fold	AF >0.66, coverage depth >22, a quality of call >7, and a window size of 12 bps	Basequal >13, min cov >10, minfrac >0.9
Remark	See publication [16]	See next chapter	See snippy [12]

To establish a reliable gold standard, we used SNP calls generated by snippy applied to Illumina short-read data from the same bacterial strains. Snippy is widely used in bacterial genomics, particularly for public health investigations of outbreaks, as it provides accurate, consistent SNP calls from Illumina sequencing data [4,9, 43].

As ONT sequencing data can be erroneous and clair3 was developed specifically for human data, we optimized different parameters influencing the variant calling process to achieve the best possible performance of clair3. These filtering parameters were selected based on their direct impact on data quality, detection sensitivity and control over potential sources of error or variant bias. Hence, the parameters quality of reads, length of reads, mapping quality, quality of SNP calls, allele frequency (reflecting the proportion of reads supporting a given variant), coverage depth, strand bias (indicating an imbalance in variant-supporting reads between the forward and reverse strands) and window size (excluding SNPs within a window around a given SNP, because several SNPs clustering within a small window can reflect one underlying evolutionary event rather than multiple separate mutations) were optimized with the help of the F1 score [Formula (3)] for each species. Afterwards, the remaining parameters impacting the F1 score were optimized together across species using the Adjusted Rand Index (ARI) and the F1 score [Formula (5)] for clair3 and as reference the SNPs derived from the Illumina data in combination with snippy. The ARI assesses the similarity between two clusters, correcting for chance agreement, and is sensitive to both the number of clusters and the assignment of individual isolates to those clusters, making it well-suited for evaluating epidemiologically meaningful groupings.

Afterwards, each analysis strategy was initially evaluated for its ability to correctly identify SNPs relative to Illumina-based snippy calls. Specifically, we calculated precision [Formula (1)], recall [Formula (2)] and F1 score [Formula (3)] for each method. While SNP calling accuracy is important in outbreak investigations, it is ultimately critical that the called variants yield reliable isolate clustering. Therefore, we extended our evaluation to examine the impact of SNP calling accuracy on downstream clustering analyses. From the results obtained by each method, we generated cgSNP alignments and applied hierarchical clustering to group isolates. We then compared these clustering results with the clustering obtained from the Illumina-based snippy calls, which served as the reference. To quantify clustering agreement, we used the ARI [Formula (5)].

### Optimal parameter setting for clair3

The optimization results for each parameter across the four species indicated that the quality of read, read length and mapping quality had no significant impact on the F1 score (Fig. S1a–c). In contrast, strand bias correction, so excluding positions with disagreeing bases in the forward and reverse read, negatively affected performance, reducing the F1 score (Fig. S1g). Focusing on the remaining parameters, allele frequency, coverage depth, quality of call and window size, a comprehensive optimization (considering the F1 score and the ARI) across all species was performed to identify a robust, globally optimal configuration. The best-performing set of parameters was: an allele frequency (AF) >0.66, a depth (DP) >22, a quality of call >7 and a window size of 12 bps (Fig. S2).

### Assembly-based snippy approach achieves the highest F1 score

**Fig. 2.**
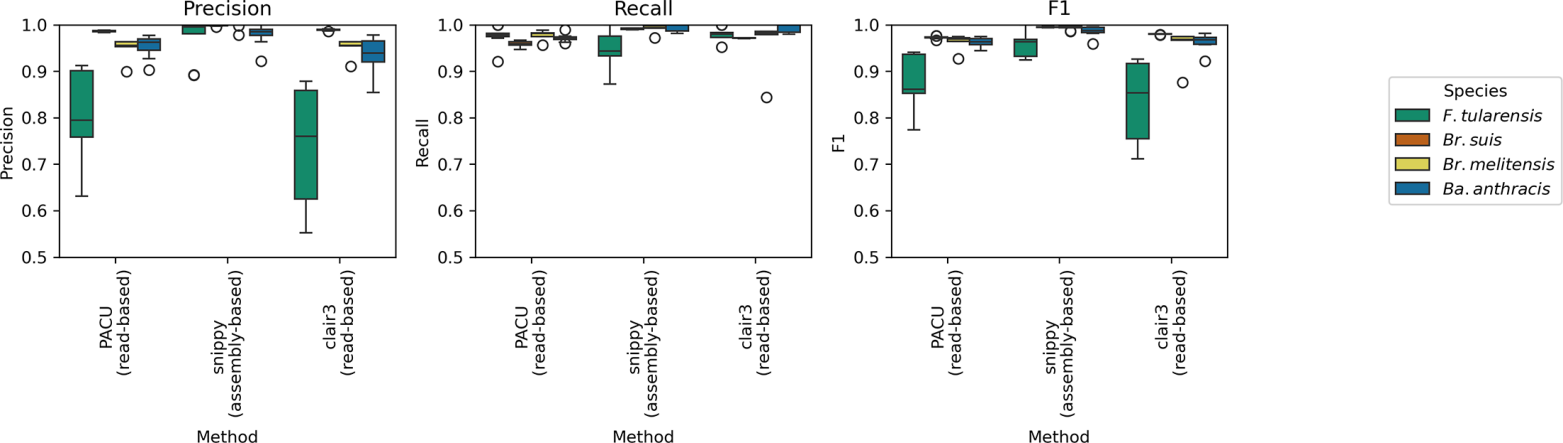
Precision, recall and F1 for PACU, clair3 and snippy with assemblies for *Ba. anthracis*, *Br. melitensis*, *Br. suis* and *F. tularensis*. Boxplots show the results for the respective strains of each species. As reference the SNPs identified using Illumina reads and snippy were taken.

Next, we compared three SNP-based analytical strategies for ONT sequencing data: PACU (read-based), clair3 (read-based, with parameters from optimization) and snippy (assembly-based, with generated artificial reads). For each strain, we evaluated SNP calling performance in terms of precision, recall and F1 score (Fig. 2).

SNP calling performance varied notably across methods and species. The PACU pipeline (read-based) demonstrated strong performance overall, particularly for *Ba. anthracis*, where it achieved a precision of 0.953, a recall of 0.972 and an F1 score of 0.962. High accuracy was also observed for *Br. melitensis* (precision 0.951, recall 0.978 and F1 score 0.9643) and *Br. suis* (precision 0.986, recall 0.960 and F1 score 0.973). For *F. tularensis*, PACU showed moderate precision (0.802) and high recall (0.976), yielding an F1 score of 0.877. The reduced precision in *F. tularensis* is attributable to a high number of false positives, averaging ~90 per strain, in genomes with ~485 true variants per strain (Table S2).

Clair3 (read-based) displayed variable performance across species. For *Ba. anthracis*, clair3 yielded a precision of 0.9351, a recall of 0.9931 and an F1 score of 0.9627. For *Br. suis* and *Br. melitensis*, clair3 achieved high precision (0.9888 and 0.9527, respectively) and maintained robust recall (0.9714 and 0.9658), resulting in consistently high F1 scores (0.9801 and 0.9590). In contrast, for *F. tularensis* clair3 exhibited the weakest performance, with precision at 0.7359 and recall at 0.9804, yielding an F1 score of 0.8345. This decline resulted from a high number of false positives. Clair3 called on average 476 true positives per *F. tularensis* strain but also falsely called on average 117 false positive variants, leading to reduced precision (Table S2). Between 88 and 100% of the removed SNPs (correctly and incorrectly) were caused by the window size parameter (Table S2).

Snippy applied to polished ONT assemblies consistently achieved the highest accuracy across all species. For *Ba. anthracis*, precision reached 0.9773, recall 0.9941 and the F1 score was 0.9855. Similarly high performance was seen for *Br. melitensis* (precision 0.9970, recall 0.9924 and F1 score 0.9947) and *Br. suis* (precision 0.9985, recall 0.9912 and F1 score 0.9948). In *F. tularensis*, snippy also performed strongly with a precision of 0.9716, a recall of 0.9487 and an F1 score of 0.9589.

### All methods consistently cluster strains in concordance with the Illumina data, with only minor exceptions

**Fig. 3.**
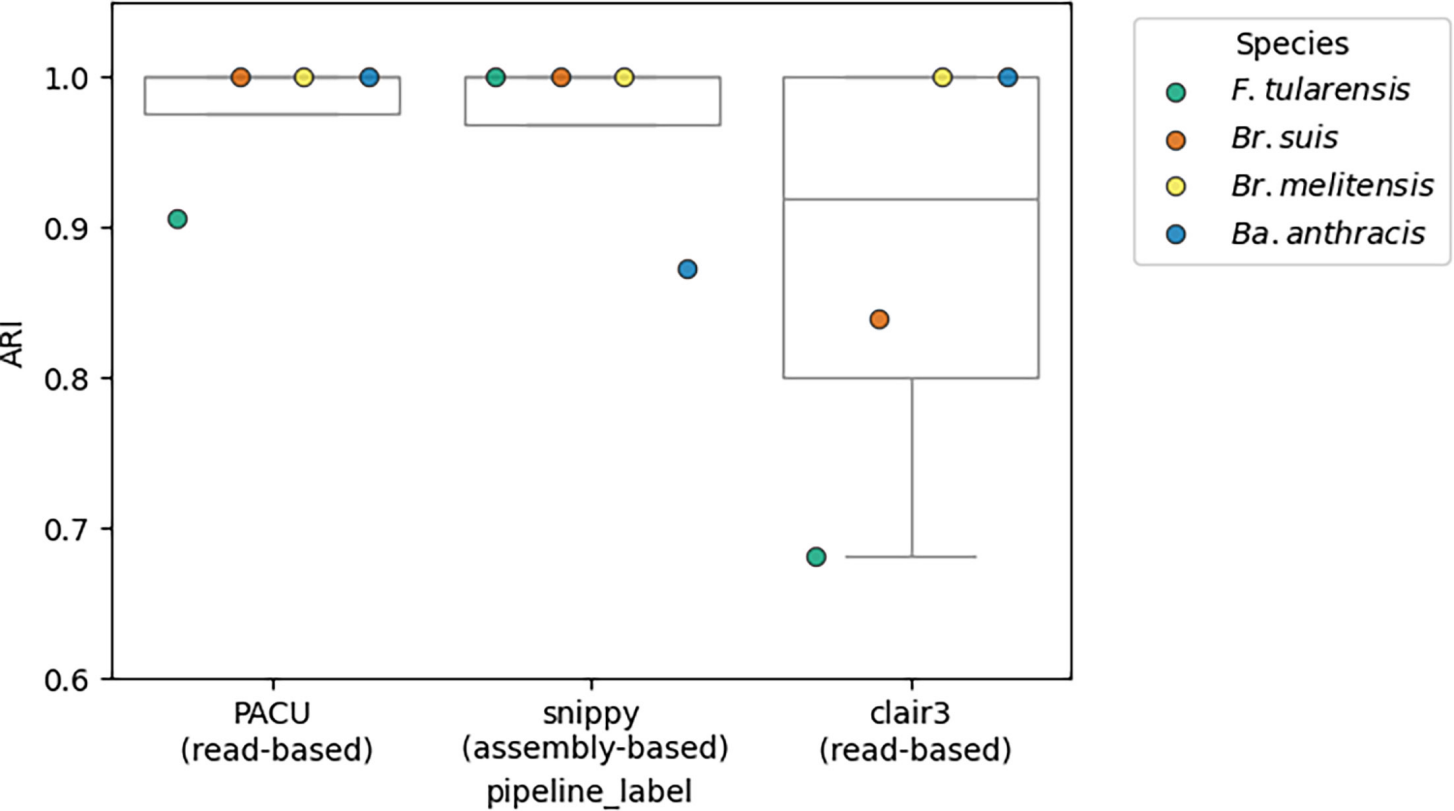
ARI per method. Boxplots summarizing the distribution of ARI scores, both ranging from 0 (no concordance) to 1 (perfect concordance), for each method across multiple species. Each point represents the score of an individual species, coloured by species identity.

For each method, we generated cgSNP alignments and computed pairwise distances between strains. These distances were used to cluster strains and infer potential outbreak groupings. Clustering results were compared to the Illumina-derived clusters with the ARI (Fig. 3).

**Fig. 4.**
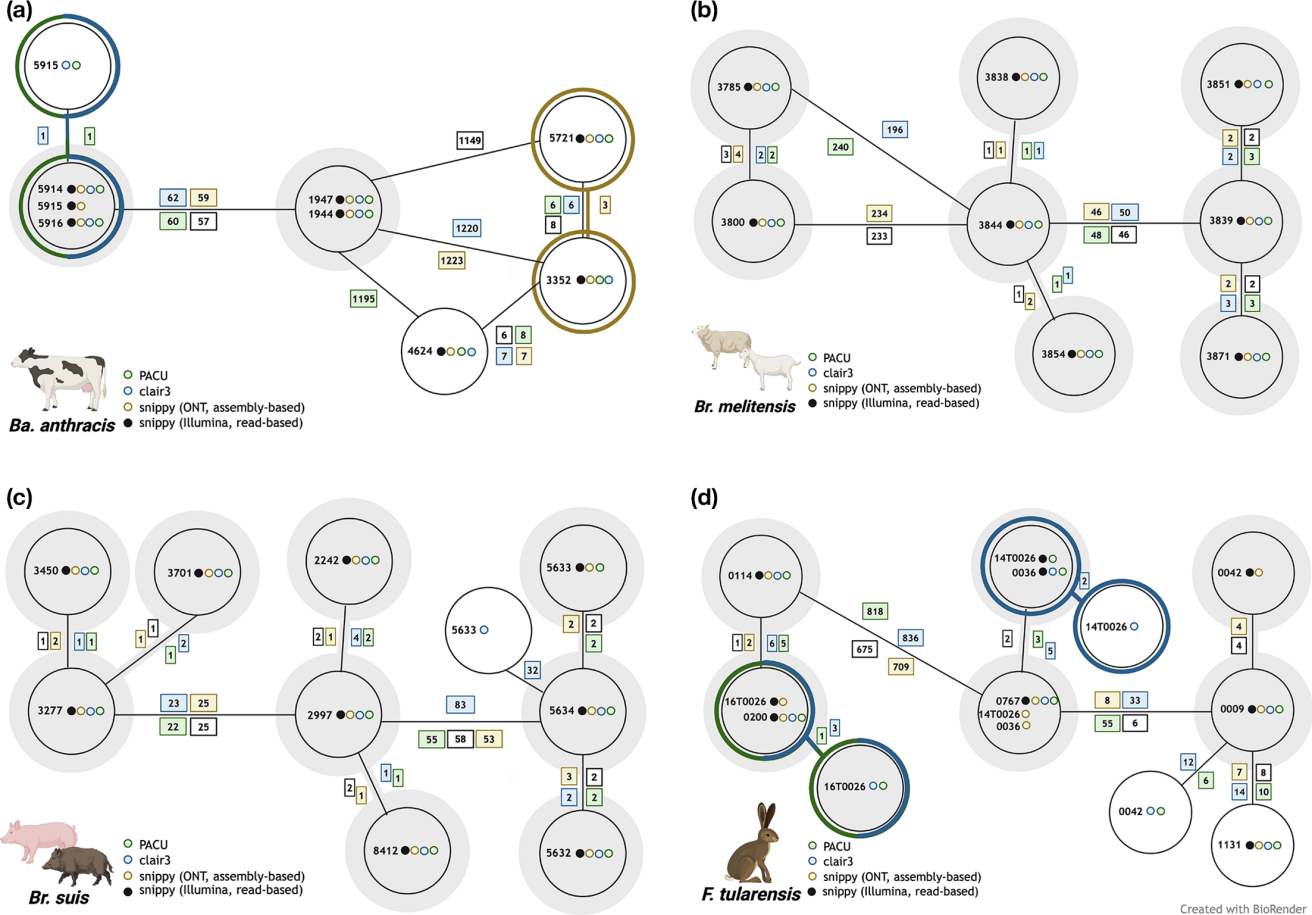
Minimum spanning tree of the cgSNP-based clustering for each species [(**a**) *Ba. anthracis*, (**b**) *Br. melitensis*, (**c**) *Br. suis*, (**d**) *F. tularensis*] for each method. Clusters were defined using a maximum threshold of five SNP differences. Grey areas indicate clusters based on Illumina data, while thick lines represent clusters derived from the corresponding method using ONT data. A dot following a strain label suggests that the strain was placed at that position by the respective method. Numbers next to the edges represent the cgSNP differences as determined by that method. Isolate names are abbreviated to their last four characters, except when this abbreviation was not unique (as for 14T0026 and 16T0026).

The *Ba. anthracis* strains from Dobichau (14RA5914, 14RA5915, 14RA5916) showed a maximum difference of one cgSNP among them across all three methods (clair3, PACU and snippy) (Fig. 3a). This is consistent with the clustering observed in the Illumina data, indicating no discrepancy between the ONT- and Illumina-based cluster assignments. Similarly, the strains isolated in Stendal (12RA1944 and 12RA1947) were identical at the cgSNP level, with no differences detected by any of the tools. This finding is also fully concordant with the Illumina data (Fig. 4a). For strains 09RA5721, 22RA24624 and 21RA23353, all originating from the same farm but isolated more than 10 years apart, we observed pairwise differences ranging from three to eight cgSNPs in the ONT data. These results are consistent with the Illumina-based analysis, which showed differences of six and eight cgSNPs. Still, snippy (ONT) results in less than five cgSNPs difference between strains 09RA5721 and 21RA23352, thus leading to the same cluster for these isolates. While these strains were clearly closely related, the observed genetic distances exceed the clustering threshold of five cgSNPs for the Illumina data (Fig. 4a). As a result, they did not fall into the same SNP cluster despite their shared origin and close relationship among Illumina data, PACU and clair3. Consequently, the reduction in ARI for snippy based on polished assemblies [PACU: 1.0, clair3 : 1.0 and snippy (ONT): 0.87, Fig. 3] was caused by the misclustering of the 09RA5721–22RA24624–21RA23353 group. Snippy’s assembly approach fails to call 15 true variants for 09RA5721 (Table S2). Those missing calls were sufficient to shrink the observed cgSNP distance beneath the five SNP threshold, causing the same cluster merging.

**Fig. 5.**
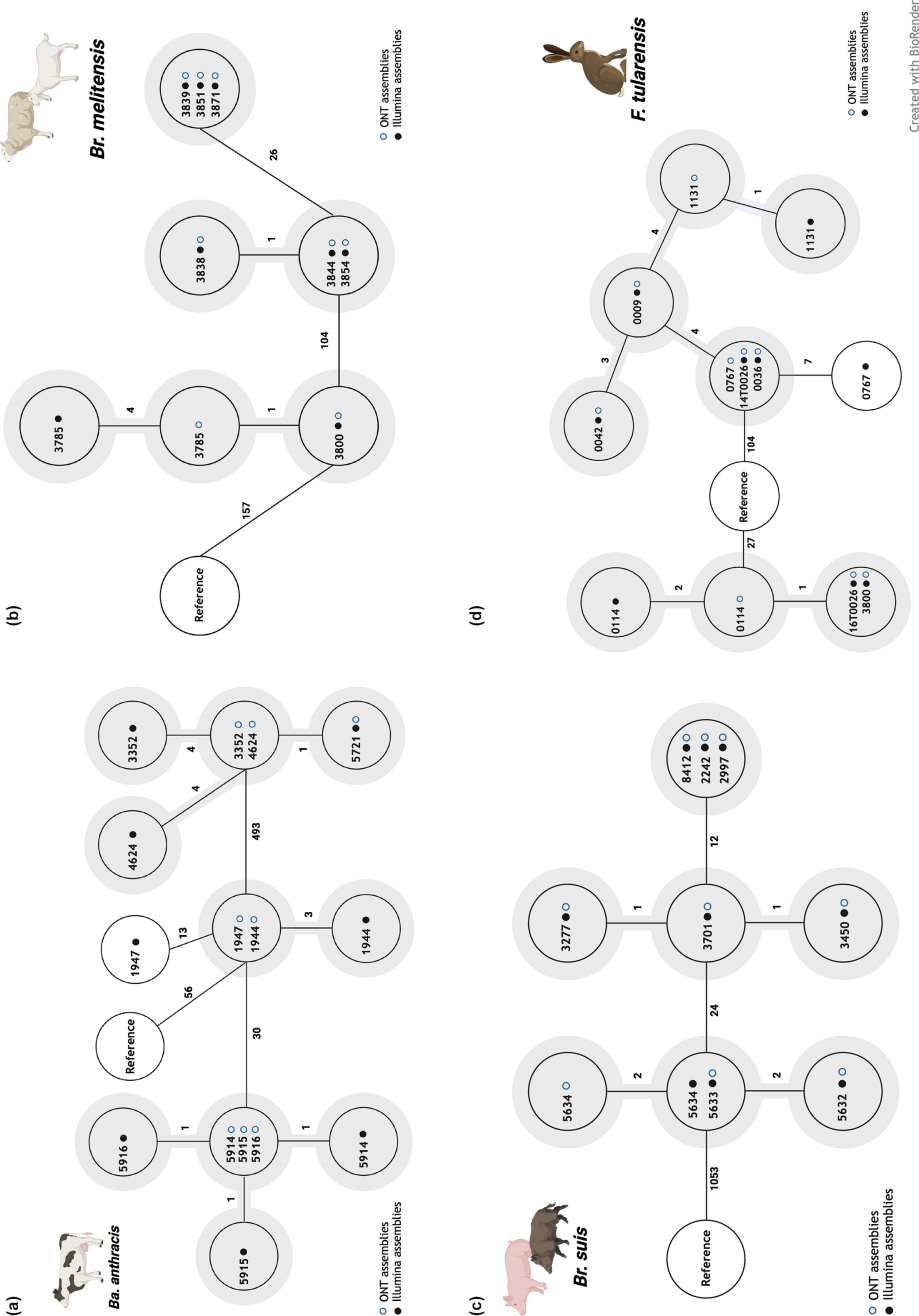
Minimum spanning tree of cgMLST profiles for the four species. Dark blue indicates the ONT-based assembly of each strain, while black represents the Illumina-based assembly. cgMLST analysis was performed using SeqSphere+, with clusters defined by a maximum of five allelic differences. Clusters are highlighted in grey, and each branch label indicates the number of allele differences.

For *Br. melitensis*, strains 21RB23785 and 21RB23800 showed a difference of one cgSNP using PACU and clair3, while snippy resulted in two cgSNP differences compared to the Illumina data (Fig. 5b). Despite these slight variations, all methods consistently placed the strains within the same cluster (as the cutoff is five cgSNPs), in agreement with the clustering observed in the Illumina-based analysis. For strains 21RB23838, 21RB23844 and 21RB23854 from Istanbul Pendik, Maras and Sanliurfa, clair3, PACU and snippy (with assemblies) detected up to two cgSNP differences between the strains, placing them all within the same cluster. This clustering is consistent with the Illumina data, confirming their close genetic relatedness across all methods (Fig. 4). Strains 21RB23839, 21RB23871 and 21RB23851 differed by up to three cgSNPs across all three methods (clair3, PACU and snippy using polished assemblies), placing them within the same cluster. This clustering was consistent with the Illumina data for all strains (Fig. 4b). All three methods correctly clustered all strains in full concordance with Illumina data, leading to an ARI score of 1.0 (Fig. 3).

For *Br. suis*, strains 08RB3450, 08RB3701 and 08RB3277, all isolated from Ludwigslust-Parchim in 2008, clustered together, with a maximum difference of two cgSNPs across all methods used. This clustering pattern was in concordance with the Illumina data, where the strains also differed by no more than two cgSNPs, confirming their close genetic relationship across both sequencing platforms (Fig. 4c). Strains 15RB2997, 14RB8412 and 15RB2242 differed by up to three cgSNPs across all ONT analysis methods. Despite these small differences, all tools consistently assigned the three strains to the same cluster. This clustering was comparable to that in the Illumina data, where the strains differed by up to two cgSNPs, confirming overall concordance between the two sequencing platforms (Fig. 4c). For strains 17RB15632, 17RB15633 and 17RB15634, the clustering results vary between methods (Fig. 4c). Using PACU and snippy with assemblies, all three strains clustered together with up to five cgSNP differences, consistent with Illumina data where they clustered together with up to four cgSNP differences. However, clair3 showed a different pattern: strains 17RB15632 and 17RB15634 clustered together with two cgSNP differences, but strain 17RB15633 differed significantly from the other two, with 24 and 32 cgSNPs differences, respectively. This suggested a discrepancy in clustering for strain 17RB15633 when analysed with clair3 compared to the other methods and Illumina data. Both PACU and snippy achieved perfect clustering in line with the Illumina data. The drop in ARI for clair3 was caused by strain 17RB15633, which exhibited much higher cgSNP differences than observed with the other tools or Illumina data. Therefore, it was excluded from its expected cluster, leading to an ARI score of 0.84 (Fig. 3).

For *F. tularensis* strains 13T0036, 14T0026 and 15T0767 from Ludwigsburg, PACU and snippy analyses on assembled ONT data showed up to three cgSNP differences among the strains. This was in line with Illumina data, resulting in up to two cgSNPs separating these strains (Fig. 5d) . Hence, these methods consistently cluster these strains together. Clair3 resulted in only 14T0026 and 13T0036 clustering together with two cgSNPs difference. 15T0767 differed in seven cgSNPs exceeding the threshold for clusters of five cgSNPs (Fig. 4). For strains 14T0114, 16T0026 and 18T0200, cgSNP analysis revealed varying levels of genetic distance depending on the method used (Fig. 4d). Snippy, based on assembled ONT data, detected only two cgSNP differences among the three strains. PACU analysis showed greater variability, with differences of up to six cgSNPs observed between the strains, leading to exclusion of 14T0114 from the cluster. Clair3 analysis revealed a divergence between strains 14T0114 and 18T0200, with six cgSNPs separating them, leading to the exclusion of strain 14T0114 from the cluster. Strain 19T0042 differed from 13T0009 by four cgSNPs according to snippy assembly analysis, consistent with the Illumina data, where they also cluster together. However, PACU reported a difference of six cgSNPs and clair3 showed 12 cgSNPs between these two strains, resulting in them not clustering together based on those methods. Strain 17T1131 differed from 13T0009 by 7 cgSNPs (snippy ONT), 10 cgSNPs (PACU) and 14 cgSNPs (clair3). Illumina data reported eight cgSNP differences for this strain. Consequently, 17T1131 did not cluster neither with 19T0042 nor 13T0009, consistently across all methods. The exclusion of two strains by PACU and three strains by clair3 led to reduced ARI scores of 0.90 and 0.68, respectively (Fig. 3).

### High concordance between ONT and Illumina assemblies was observed for *Brucella*, whereas assemblies for *Ba. anthracis* and *F. tularensis* demonstrated greater variability

The cgMLST analysis of *Ba. anthracis* demonstrated high concordance between ONT- and Illumina-based assemblies (Fig. 4a). Among the eight strains analysed, only one strain showed identical cgMLST profiles between Illumina and ONT assemblies. Two strains differed by a single locus, two strains had four loci differences each, one strain exhibited a three loci difference, and one strain (12RA1947) showed a 13 loci difference. Reasons for these differences were local low coverage by the Illumina data (locally <22 fold), as well as homopolymeric regions. In detail, strain 12RA1947 with 13 loci difference in the Illumina assembly was excluded from its corresponding cluster. Here, especially coverage of the Illumina reads ranged from 6 fold to 22 fold within most of the differing loci (12 loci), while the ONT data covered the loci much higher (> 470 fold).

The cgMLST analysis of *Br. melitensis* demonstrated strong concordance between ONT- and Illumina-based assemblies (Fig. 5b). Except for one strain, 21RB23785, all assemblies clustered identically regardless of sequencing technology, indicating no differences in their cgMLST loci attributable to the platform used. The analysis clearly delineated the three distinct clusters, which were consistently resolved across both sequencing methods. 21RB3785 differed at four cgMLST loci between the Illumina and ONT assemblies, due to low local coverage (11–36 fold for ONT and 17–38 fold for Illumina).

The cgMLST analysis of *Br. suis* showed near-complete concordance between Illumina and ONT assemblies (Fig. 5c). All strains clustered identically across both sequencing methods, except for one strain, which showed a difference of two cgMLST loci between the ONT and Illumina assemblies, both of which were linked to a homopolymeric region. Despite this minor discrepancy, all three clusters were clearly resolved regardless of the sequencing technology used.

The cgMLST analysis of *F. tularensis* revealed some discrepancies between Illumina- and ONT-based assemblies (Fig. 5d). For two strains, 14T0114 and 17T1131, the assemblies differed by one and two cgMLST loci when comparing ONT and Illumina, caused by low coverage (<10 fold for Illumina). More notably, one strain, 15T0767, exhibited seven differing cgMLST loci between the Illumina and ONT assembly. Here, low coverage (locally 7 –20 fold for Illumina) as well as homopolymeric regions were responsible for these differences. This strain was particularly important because the Illumina assembly is placed outside the cluster, suggesting it is unrelated, whereas the ONT assembly was grouped within the cluster, indicating it belongs to the cluster.

## Discussion

In this study, the performance of ONT sequencing for high-resolution genotyping of four bacterial pathogens, *Ba. anthracis, Br. melitensis, Br. suis* and *F. tularensis* was evaluated using both SNP-based and cgMLST-based clustering approaches. By benchmarking ONT-derived results against Illumina sequencing data, the accuracy and epidemiological reliability of three SNP-based analysis strategies (PACU, clair3 and snippy based on polished assemblies) and cgMLST analyses were assessed. In light of the controlled strain propagation described in the Methods section, and the low mutation rates reported for these species under laboratory conditions, the discrepancies observed between Illumina and ONT datasets are more likely attributable to sequencing-related errors than to true biological variation.

### Optimization of filtering parameters

The optimization of parameters for clair3 revealed trade-offs between precision, recall and overall SNP calling accuracy. The best combined performance (F1=0.936, precision=0.906, ARI=0.881) was achieved with thresholds that retained a broader set of variants, including those with lower but consistent support. A comparative analysis with a recent study optimizing bcftools filtering parameters further contextualizes these results [16]. That study, using *Escherichia coli* and *Listeria* (L*.*) *monocytogenes*, both well characterized and relatively fast mutating bacteria, identified optimal parameters of allele frequency=66, coverage depth=5, quality of call=50 and window size=10, with a focus on maximizing precision. Notably, although the types of parameters overlap with those influencing the accuracy of SNP calling with clair3 in this work, the threshold values differ (e.g. our optimal coverage depth=22, quality of call=7), suggesting that the variant caller (clair3, which utilizes neural networks, versus the heuristic based bcftools), and organism-specific biology both influence optimal parameter settings. Both studies examined strand bias as a potential parameter, but neither observed improvements in variant calling performance upon its inclusion. This is somewhat surprising, given that multiple prior investigations of cgMLST performance have identified methylation as a significant source of errors [15,23]. Methylation-induced artefacts often manifest as ambiguous bases in regions flanking methylated sites, typically because errors occur on only one strand of the DNA molecule. One would therefore expect strand bias filtering to help mitigate these errors; however, our results and those of others’ [16] indicate that filtering on strand bias either fails to improve or even reduces overall SNP calling accuracy.

### Accuracy of SNP calling

Across the evaluated SNP-based analysis approaches PACU, clair3 and snippy, consistent trends in performance were observed, with snippy (based on polished assemblies with generated artificial short reads) generally achieving the most balanced precision and recall, followed closely by PACU, and then clair3. While snippy (based on polished assemblies) yielded comparably high performance across all species, clair3 and PACU showed variability, particularly struggling with *F. tularensis*.

Snippy, when applied to polished ONT assemblies, maintained consistently high precision and recall across all organisms. In fact, Snippy’s assembly approach retains nearly all true SNPs and excludes most spurious ones, which explains its superior low false-positive rate. This high performance may be further supported by the methodological consistency of using Snippy for both the Illumina reference and the ONT-based calls, as shared algorithmic heuristics likely minimize tool-specific discrepancies. Furthermore, unlike long read-based tools, snippy uses high-quality assemblies to generate short reads in silico and maps these short reads to the reference. That way, noise in ONT’s long raw reads is being bypassed. Long ONT reads are prone to systematic errors (e.g. in homopolymer regions) and polishing during and post-assembly with tools like medaka or clair3 reduces these errors. While assembly-based SNP calling with snippy is a particularly effective strategy for ONT data, it clearly depends on the use of high-quality assemblies. Assemblers have to be chosen carefully, as the performance was shown to differ between different species [44]. It can be expected that assembly-based SNP calling yields slightly different results when using different assembly approaches, even with the same raw data.

PACU displayed consistently high precision, recall and F1 scores for *Ba. anthracis* and *Brucella* spp., suggesting robust detection capabilities. For *F. tularensis*, however, precision dropped. PACU’s filtering strategy is intentionally conservative, discarding variants with an allele frequency below 0.66 or a coverage depth below five. This effectively reduces false positives in noisy datasets but can also lead to missed SNPs, which was not observed extensively in this study. Its filters generally perform well, but occasionally retain borderline SNPs, leading to false positives, as happened for *F. tularensis*. Additionally, PACU was optimized using *E. coli* and *L*. *monocytogenes*, both of which are part of the ONT basecalling training sets [45], which likely explains why these species yield better results than those not represented in the training data.

Clair3 has been noted for its high precision in SNP calling by Hall *et al*. [24], who evaluated the tool using a combination of common bacterial species and a pseudo-real approach where synthetic variants were introduced into the data. In contrast, our evaluation using real-world data from rather less studied bacterial species regarding ONT sequencing revealed that while clair3 maintained high precision and recall for *Ba. anthracis* and *Brucella* spp., it showed notably lower recall and increased false positive rates in *F. tularensis*. This discrepancy could be attributed to clair3’s deep learning model, which, despite its power, may be less effective when applied to exotic or low-diversity bacterial genomes likely due to a training bias favouring common pathogens and synthetic variant scenarios rather than real-world genomic variation.

### Clustering and outbreak detection

Accurate outbreak reconstruction is a key goal of genotyping methods. Accuracy of clustering was studied using the ARI. Here, snippy (based on polished assemblies) achieved the highest scores, followed by PACU and then clair3. For some species (*Br. melitensis, Br. suis*), all methods performed equally well, with nearly perfect clustering. However, discrepancies arose in *Ba. anthracis* and *F. tularensis*, where some methods under-clustered (false-positive merges) or over-clustered (false-negative splits) due to minor SNP count deviations. While a threshold of five cgSNPs has been shown to provide a good fit between genomic clusters and epidemiological data, such fixed cut-offs should be regarded only as rules of thumb, and cluster relaxation may be necessary depending on the biological context and epidemiological situation.

For *Ba. anthracis*, subtle differences in calling sensitivity have profound impact on outbreak cluster assignments for the strains 09RA5721, 21RA23352 and 22RA24624. While for all other methods these strains remain singletons, for snippy (assembly-based) strains 09RA5721 and 21RA23352 differ only in three cgSNPs, leading to clustering together. Snippy (assembly-based), despite its generally high performance when applied to ONT assemblies, misses to call 15 true SNPs. These missing calls alone are sufficient to reduce the observed cgSNP distance between isolates, again resulting in erroneous clustering. This discrepancy may be explained by the unusually high divergence between this strain and the reference genome used, with ~1,200 SNP differences compared to ~100 SNPs for other strains. Bush *et al*. [11] showed that the degree of reference similarity is a key factor influencing SNP calling accuracy in Illumina data, with higher divergence leading to lower recall due to misalignment and missed variant calls. Importantly, Wick *et al*. [46] extended these observations to ONT data, demonstrating that SNP calling from ONT assemblies also suffers reduced accuracy as variant density increases.

The false clustering of *F. tularensis* strains by PACU and clair3 was primarily driven by elevated false-positive rates, which inflated pairwise SNP distances and led closely related strains to exceed the clustering threshold. A major contributor to these false positives was miscalling in homopolymer regions. In fact, 82–89% of all false positive variants in clair3 and PACU occurred within homopolymers, with over half of them located in repeats of 2 bps length. The size of the cgSNP alignments for PACU and clair3 was much larger with 900–920 SNPs, compared to ~640 SNPs when using snippy on ONT-based assemblies. While snippy on Illumina data was used as reference many false positives in clair3 and PACU corresponded to positions excluded or filtered out in the Illumina-based calls and not being integrated into the ONT assemblies. Hence, the assembly-based snippy approach maintained very high precision (>98%) and did not show these effects, leading to more accurate clustering and shorter, cleaner alignments.

It was shown that PACU can produce reliable SNP calls and accurate clustering for multiple bacterial species, despite the inherent error rates of ONT data. This aligns with findings from the study, where PACU was published [16], in which PACU successfully reconstructed phylogenetic trees for *L. monocytogenes* and *E. coli* based on ONT data. Their results reinforce our conclusion that PACU is a robust read-based method for SNP analysis, even in species with varying genome complexity or lower variant density. However, our study also highlights the challenges in accurately resolving closely related strains with very few SNPs, especially in *F. tularensis*. An additional disadvantage is that PACU is only applicable to a single contig reference. Therefore, reference genomes with multiple contigs from bacterial species with more than one chromosome must be concatenated.

When comparing our findings to those of Linde *et al*. [9], who analysed a subset of overlapping strains for *Ba. anthracis* and *Br. suis*, some differences in results become apparent. For *F. tularensis*, Linde *et al*. reported no more than three cgSNP differences between variant calls derived from snippy applied to ONT assemblies and those from Illumina data. Our study observed similarly high concordance between snippy-based analyses on ONT assemblies and Illumina reads across a distinct set of *F. tularensis* strains. However, ONT read-based SNP callers demonstrated greater variability in cgSNP distances, though these approaches were not evaluated in the Linde *et al*. study. In contrast, *Brucella spp*. presented a different pattern. While Linde *et al*. observed between 20 and 70 cgSNP differences among *Br. suis* strains using earlier ONT flowcells (R9.4.1 and R10.4.0), our results, based on the latest flowcell technology (R10.4.1) and bioinformatic tools, show near-perfect clustering with minimal differences between ONT and Illumina for both *Br. suis* and *Br. melitensis*, including the identical strains common to both studies. This improvement indicates that the previously observed errors in *Brucella* variant calling have been largely mitigated with the newer flowcell chemistry. Moreover, updated bioinformatics tools and base calling models further improved the accuracy of SNP calling for *Br. suis*. Notably, one *Br. suis* strain (17RB15633), not included in Linde *et al*., showed significant divergence. These 32 cgSNPs, which all lay in the same region NC_004311.2 : 408163–426926, were false positives. Of these, 56% are located in homopolymeric regions, when called with read-based clair3. This indicates a specific clair3-related problem with such regions. For *Ba. anthracis*, our findings largely replicated those of Linde *et al*. for the identical strains used in both studies.

### Core genome multilocus sequence typing

CgMLST analyses revealed high concordance between ONT and Illumina assemblies for *Br. melitensis* and *Br. suis*. However, greater discrepancies were noted in *Ba. anthracis* and *F. tularensis*, where ONT assemblies diverged from Illumina-based profiles by up to 13 alleles in two cases. Despite these differences, all outbreak clusters could be recovered across both platforms, except for two outliers, one *Ba. anthracis* and one *F. tularensis* isolate, that fell outside the clustering threshold in the Illumina assemblies.

Minor divergences observed between ONT and Illumina assemblies across all strains are likely due to a combination of known technical limitations. One major factor is the presence of homopolymers, which are a well-documented source of basecalling and assembly errors in ONT sequencing [44,47], as discussed in the preceding section of this study. Interestingly, Hall *et al*. [46] note that even high-depth ONT data with advanced basecalling can still result in false positives, especially in repetitive or homopolymeric regions, which may explain some of the cgMLST discrepancies that were observed. In addition, low coverage in either ONT or Illumina datasets can lead to incomplete or incorrect allele assignments, further contributing to discrepancies in cgMLST profiles. While these differences were generally minor, they highlight the importance of optimizing both sequencing depth and quality, especially when high-resolution genotyping is required.

In contrast to the findings of Linde *et al*. [9], who reported higher cgMLST variability in ONT assemblies of *Br. suis* compared to *F. tularensis*, our study showed full concordance between ONT and Illumina data for *Br. suis,* except for one strain with minor differences (two alleles). This improvement may reflect differences in the specific strains analysed, as well as advancements in ONT technology, such as improved flowcells, basecalling neural networks or chemistry, that have enhanced sequence accuracy since the previous study.

The *Ba. anthracis* and *Br. suis* strains included in both our study and that of Linde *et al*. [9] clustered identically in both datasets, with matching allele differences. Importantly, while the Illumina data used for these strains were the same in both studies, the ONT data was newly generated using each three separate flowcells of the newest chemistry. This consistency suggests that the observed clustering patterns are not artefacts of ONT sequencing. Instead, it points to two plausible explanations. Either Illumina assemblies are less precise in resolving certain cgMLST loci, perhaps due to locus drop-outs or misassemblies, or, less likely, a systematic ONT-specific error has persisted across all sequencing runs and flowcell versions. Given the technological improvements in ONT and the independent nature of our sequencing efforts, the former explanation seems more probable, further supporting the reliability of ONT for high-resolution genotyping of *Ba. anthracis* and *Br. suis*. It has to be noted that also Illumina technology has its limitations, as it shows inherent problems, like uneven coverage, particularly in G+C-rich DNA regions [48]. Further, the quality depends on the approach (paired- vs. single-end) as well as chemistry used for sequencing.

Notably, the two isolates, one *F. tularensis* and one *Ba. anthracis*, that differed by more than the five allele cgMLST clustering cut-off in one of the assembly types. In both cases, it was the Illumina based assemblies that fell outside the threshold, whereas the ONT profiles remained within five alleles of their respective outbreak clusters. When mapped back to a high-resolution SNP phylogeny, these two strains clearly group with their presumed outbreak partners, suggesting that the ONT data provided the more accurate clustering. This finding shows that some of the apparent cgMLST discrepancies may result from locus drop-outs or misassemblies in short-read data rather than inherent limitations of ONT sequencing.

Our findings align with several recent studies demonstrating that cgMLST based on ONT data can yield results highly concordant with Illumina sequencing across multiple bacterial species. For example, a study reported full concordance for *Enterococcus* spp. and *C. diphtheriae*, with strain-specific discrepancies in *Enterococcus* linked to methylation [20]. Similarly, Biggel *et al*. [49] found cgMLST agreement in 66 of 74 *L*. *monocytogenes* isolates, with methylation again suspected in the discordant cases. A larger multicentre evaluation further supported this pattern, observing high overall concordance but methylation-associated typing errors in *Enterococcus faecium, K*. *pneumoniae, L. monocytogenes* and *Staphylococcus aureus* across ~80 isolates [23]. Lohde *et al*. [15], using a well-characterized *K. pneumoniae* outbreak, also documented basecalling errors near methylation sites that impacted cgMLST results. In contrast, a study using the newest ONT chemistry for *Bordetella pertussis* showed minimal differences across 40 strains [14]. Notably, no methylation-associated discrepancies were observed in the presented dataset, suggesting that cgMLST is robust for *Ba. anthracis, Br. melitensis, Br. suis* and *F. tularensis* under the current sequencing conditions. Nevertheless, as highlighted by multiple studies, methylation-induced errors remain a known risk in ONT-based genotyping and should be considered when interpreting results, particularly in methylation-rich genomes.

## Conclusion

We addressed the critical question of whether ONT data can be used to accurately detect and delineate outbreaks using SNP calling and cgMLST approaches. For four low-mutation-rate BSL-3 species and multiple epidemiologically defined outbreaks, ONT-based results were evaluated against epidemiologically supported outbreak structures, using Illumina data as a high-quality reference comparator. Among the tested SNP-based analysis strategies, snippy (based on polished ONT assemblies) achieved the highest scores in precision, recall, F1 score and cluster congruence. In contrast, PACU and clair3 produced a higher number of false positives for *F. tularensis*, though they performed well across the other tested species. Most errors occurred in homopolymeric regions, highlighting the persistent weaknesses of ONT sequencing in read-based methods. Despite some minor misclusterings, read-based variant callers still delivered good clustering results. Notably, cgMLST analysis using ONT data performed at least as well as Illumina data.

## Supplementary material

10.1099/mgen.0.001742 Supplementary Material 1.

10.1099/mgen.0.001742 Table S1.

10.1099/mgen.0.001742 Table S2.

10.1099/mgen.0.001742 Table S3.

## Abbreviations

AF: allele frequency
ARI: Adjusted Rand Index
BSL-3: biosafety-level-3
cgMLST: core genome multilocus sequence typing
cgSNP: core genome single-nucleotide polymorphism
DP: depth
MLST: multilocus sequence typing
ONT: Oxford Nanopore Technologies
PACU: Prokaryotic Awesome variant Calling Utility
SNP: single nucleotide polymorphisms
SNP: single nucleotide polymorphism
WGS: whole-genome sequencing
