# Supplementary material for: Genomic outbreak investigation of biosafety-level-3 pathogens using nanopore sequencing
Source: Microb Genom. 2026 Jun 30;12(6):001742. doi: 10.1099/mgen.0.001742 (PMC13317016; doi:10.1099/mgen.0.001742)
Supplement: Supplementary Material 1. [file mgen-12-01742-s001.pdf]

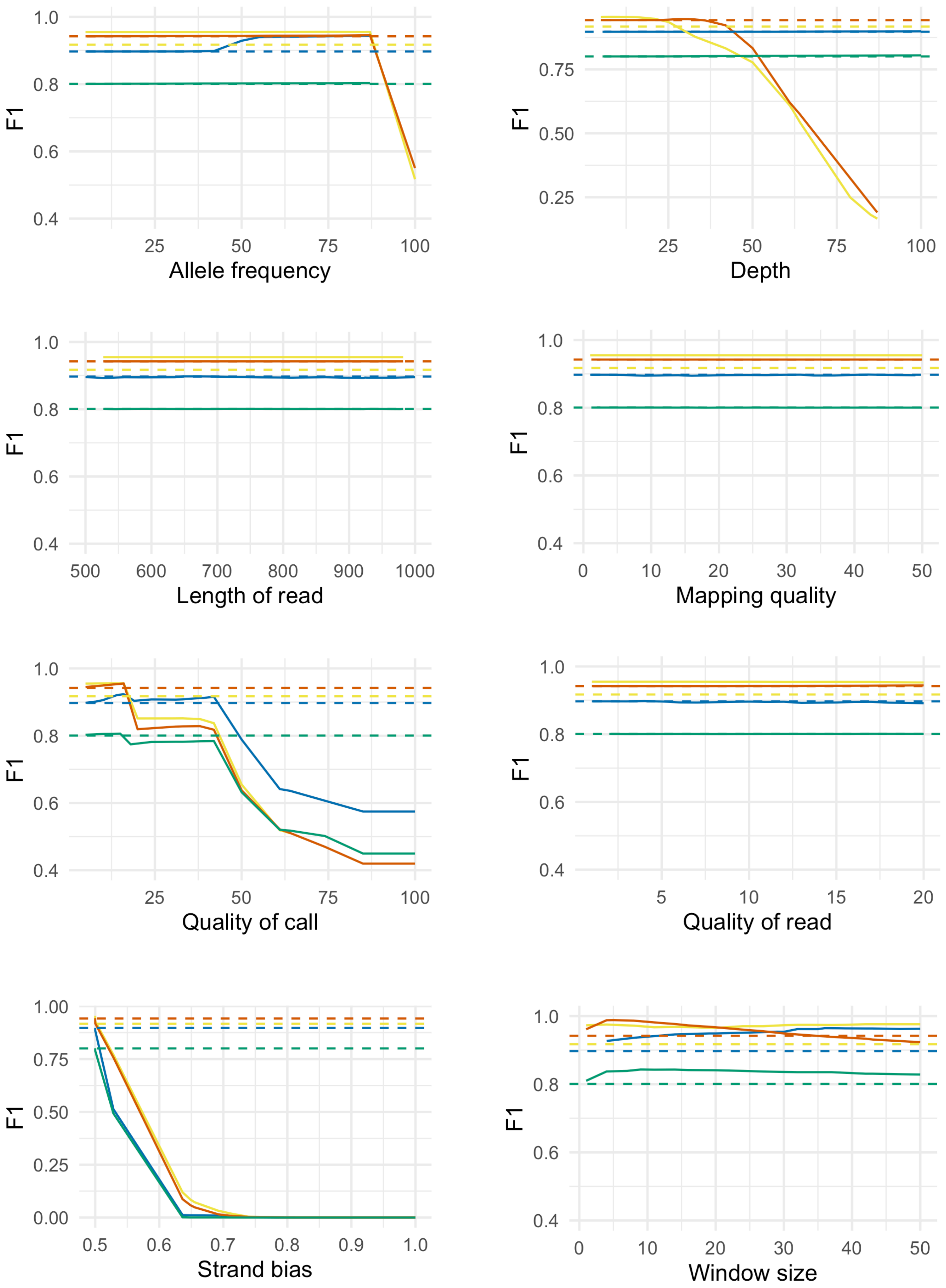

Figure S1: F1 scores for the different parameters across the different species. Each parameter was optimized for each species, with the y axis showing the F1 score and the x axis the parameter value. The dashed lines signal the F1 score without any parameter set. Colors signal the different species (*Ba. anthracis*: blue, *Br. melitensis*: yellow, *Br. suis*: orange, and *F. tularensis*: green).

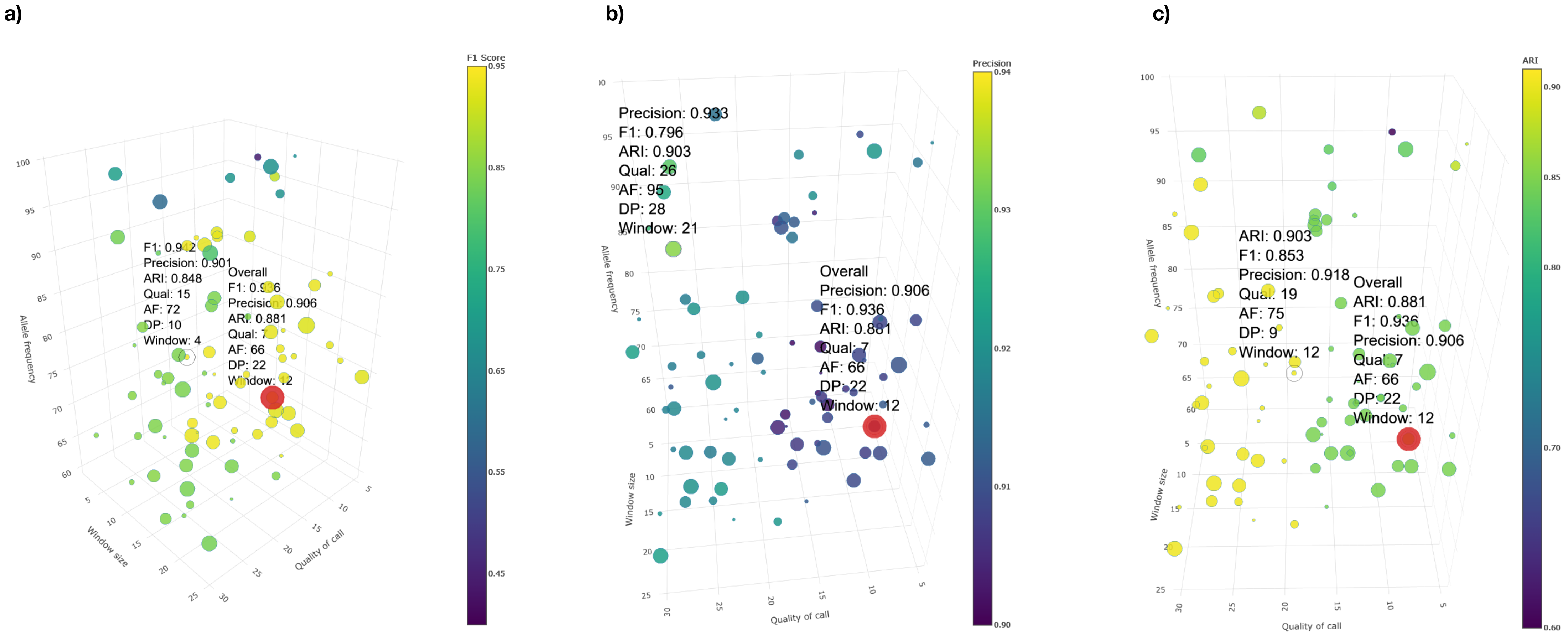

Figure S2: 3D plot of joint optimization of the parameters across all species. The axes show allele frequency, quality of call, as well as window size, while the size of the point refers to the coverage depth. The color scale visualizes the given metric (a) F1, b) precision, c) ARI). In black is the maximum score for the given metric and in red the point referring to the optimal parameters of the combined score of the optimization.
